# A Fluorescence Sensor Capable of Real-Time Herbicide Effect Monitoring in Greenhouses and the Field

**DOI:** 10.3390/s18113771

**Published:** 2018-11-05

**Authors:** Pei Wang, Hui Li, Weidong Jia, Yin Chen, Roland Gerhards

**Affiliations:** 1 Key Laboratory of Modern Agricultural Equipment and Technology, Ministry of Education, Jiangsu University, Zhenjiang 212013, China; jiaweidong@ujs.edu.cn; 2 Institute of Phytomedicine, University of Hohenheim, 70599 Stuttgart, Germany; gerhards@uni-hohenheim.de; 3 College of Engineering and Technology, Southwest University, Chongqing 400715, China; 4 Key Laboratory of Plant Protection Engineering, Ministry of Agriculture and Rural Affairs, Jiangsu University, Zhenjiang 212013, China; 5 National Center for International Collaboration Research on Precision Agricultural Aviation Pesticides Spraying Technology, South China Agricultural University, Guangzhou 510642, China; chen.3305@buckeyemail.osu.edu

**Keywords:** chlorophyll fluorescence, herbicide effect, real-time identification

## Abstract

Herbicide resistant weeds need to be identified early so that yield loss can be avoided by applying proper field management strategies. A novel chlorophyll-fluorescence-imaging sensor has been developed to conduct real-time herbicide effect evaluation. In this research, greenhouse and field experiments were conducted to calibrate the capability of the sensor in monitoring herbicide effects on different biotypes of two grass weeds (*Alopecurus myosuroides*, *Apera spica-venti*) in southwestern Germany. Herbicides with different modes of action were applied for the effect monitoring. Chlorophyll fluorescence yield of the plants was measured 3–15 days after treatment (DAT) using the new fluorescence sensor. Visual assessment of the weeds was carried out on 21 DAT. The results showed that the maximal PS II quantum yield (*F_v_*/*F_m_*) of herbicide sensitive weeds was significantly lower than the values of resistant populations in 5 DAT. The new technology was capable of quickly identifying the herbicide’s effect on plants. It can be used to optimize management strategies to control herbicide resistant weeds.

## 1. Introduction

Herbicide application is one of the most common approaches for weed control by farmers. However, unwarranted or excessive application of herbicides with the incorrect mode of action and treatment at insufficient rates have led to severe problems, such as the herbicide resistance [1].

In winter cereal fields, *Alopecurus myosuroides* and *Apera spica-venti* are considered as the most problematic herbicide resistant weeds. According to a survey by Heap [2], resistance to the inhibitors of ALS (acetolactate synthase) and ACCase (acetyl CoA carboxylase) is the main issue in winter cereal fields. Effective and rapid responses are required for identifying the resistant populations so that weed management can be carried out in a timely fashion and with proper methods. The common approach to monitoring herbicide resistance mainly depends on whole plant bioassays in the greenhouse [3]. In recent years, several modern biochemical assays like enzyme assay and PCR (polymerase chain reaction) gene sequencing techniques have been introduced for herbicide resistance monitoring in the laboratory [4]. However, intensive time, labor and financial resources are necessary during the whole assay procedure. Additionally, the above methods require the collection of seeds or plant leaves from the field. Thus, the real-time detection of herbicide effect after application cannot be provided for farmers and scientists. Therefore, in order to enable the proper management of resistant weeds in the same growth season of the crops, a more efficient herbicide resistance screening system is necessary.

Chlorophyll fluorescence imaging technology has been applied for non-destructive investigation of the physiological reaction of the photosystem II (PS II) in plants. Several studies have shown that the parameter, maximal PS II quantum yield (*F_v_*/*F_m_*) can be used to indicate the health status of the photoreaction center of the plant leaves. It has been proved that *F_v_*/*F_m_* is a sensitive parameter that indicates the biotic and abiotic stresses in plants [5]. Chlorophyll fluorescence measurement technology has been applied to differentiate weeds which are resistant or sensitive to PS II inhibitors [6,7,8,9,10]. Recently, based on laboratory research, several reports have presented the possibility of using the chlorophyll fluorescence measurement for the detection of weeds’ physiological response to the non-photosystem inhibitors [11,12,13,14,15,16,17,18]. Wang et al. [19,20] developed a mobile sensor from a chlorophyll fluorescence imaging system, which could be applied in the greenhouse and field tests. It has been applied for the early detection of the effects of herbicide on a commercially sensitive population of *Alopecurus myosuroides* and for herbicide stress detection in soybeans, a crop which is very sensitive to the tested herbicides [21,22]. However, further investigation is required to test its ability to identify herbicide resistance, for the greenhouse and field application, and on a wider range of weed species. Furthermore, in former studies, sensitive and resistant weeds were organized by commercial seed suppliers. The sensor has never been applied to diagnose weeds with unknown resistance levels.

In this study, a novel chlorophyll fluorescence-imaging sensor was implemented for real time herbicide effect monitoring in the greenhouse and the field. The second objective was to test if the sensor is capable of identifying herbicide resistant weeds in different species and different natural biotypes.

## 2. Materials and Methods

### 2.1. The Chloropgyll Fluorescence Imaging Sensor

**Figure 1:**
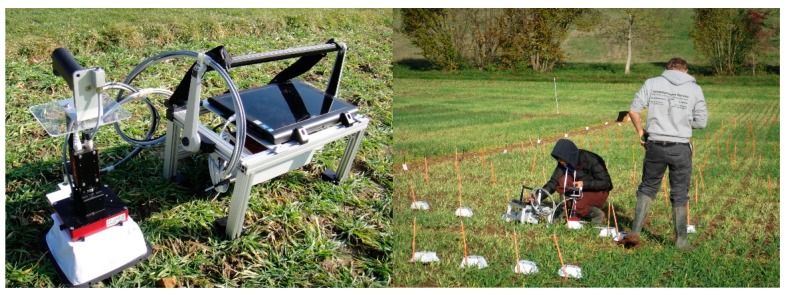
The mobile chlorophyll fluorescence sensor Weed-PAM. (**Left**): the sensor’s setup, including a camera head, a dark adaption box, a control unit with battery inside, a tablet computer and the essential cables for data communication and power supply. (**Right**): Herbicide resistance identification with the sensor in the field.

The chlorophyll fluorescence was detected using the Weed-PAM sensor by Heinz Walz GmbH (Effeltrich, Germany) (Figure 1). In the measuring head, an LED array was mounted to generate the measuring light with a wavelength of 460 nm. The measuring light was alternated with high frequency pulse to saturate the reaction center of PS II. Chlorophyll fluorescence was detected by a digital imaging chip, in front of which, a filter enabling light with wavelength longer than 620 nm was mounted. The background noise was removed by the software, “ImagingWin for Weed-PAM” as described in Kaiser et al. [11]. The software was also used to control the LED emittance switch and the frequency adaptor. It also worked as the data processing center and the fluorescence image generator. In this study, the Weed-PAM sensor was applied to detect the *F*_0_ (minimal fluorescence in the dark-acclimated state) and *F_m_* (maximal fluorescence in the dark-acclimated state) of the plants’ chlorophyll fluorescence emission. Then, the *F_v_*/*F_m_* was calculated for analysis according to the equation.

$$F_{v}/F_{m}=\frac{F_{m}-F_{0}}{F_{m}}$$

### 2.2. Greenhouse Experiment Design and Plant Preperation

#### 2.2.1. Alopecurus Myosuroides

*Alopecurus myosuroides* seeds of a sensitive population were sowed separately in the greenhouse in the Institute of Phytomedicine, University of Hohenheim, in Stuttgart, Germany. The seeds were bought from HerbiSeed (Twyford, UK). 15 × 20 cm pots filled with vermiculite were used for the seeds’ germination. The plant seedlings were transplanted into 8 × 8 cm paper pots (Jiffy, 4 plants per pot) when the first true leaf emerged from coleoptile (BBCH 10). The transferred plants were separated into four groups. Ten replicates of the pots were set up for each subgroup. The plants were treated with herbicides including ALS and ACCase inhibitors at the BBCH 22-23 growth stages. Broadway^®^ (68.3 g kg^−1^ pyroxsulam, 22.8 g kg^−1^ florasulam, Dow AgroSciences GmbH, München, Germany) and Axial^®^ 50 (50 g L^−1^ pinoxaden, Syngenta, Basel, Switzerland) were selected for ALS and ACCase treatment, respectively. Details of the herbicide treatment are presented in Table 1. The spraying volume was calibrated to 200 L ha^−1^. All the pots were placed in a completely randomized design after the herbicide application. The experiment was repeated three times from September 2015 to December 2016.

#### 2.2.2. Apera Spica-Venti

Eighty natural biotypes of *Apera spica-venti* were prepared, using the same method as mentioned above, in the greenhouse of the Plant Protection Department of Hessen, in Wetzlar, Germany. Three replicate pots of plants in each biotype were prepared for each treatment. The plants were treated with herbicides also at the BBCH 22-23 growth stages. Details of the herbicide treatment are presented in Table 1. All the pots were set in a completely randomized design after the herbicide application. The experiment was repeated two times from January to April in 2015.

### 2.3. Field Experiment Design

Field experiments were conducted at two locations, Renningen (Ihinger Hof) and Hohenheim (Hofacker), which are the main research stations of the University of Hohenheim. Seeds of herbicide sensitive *Alopecurus myosuroides* and *Apera spica-venti* (HerbiSeed, Twyford, UK) were sown separately in different fields. Herbicide application was conducted according to the same design as the greenhouse experiments. In each field, the experiment was set up as a randomized complete block design with three blocks and three treatments (including the control group). The application was carried out using a motorized plot sprayer. The experiment was repeated two times in February 2015 and March 2016.

### 2.4. Measurements

In all experiments, the *F_v_*/*F_m_* value of all plants in the greenhouse and 30 plants in the field, per treatment were measured. In the field, all the plants were selected randomly in each treatment plot. During the measurements, each measured plant was marked. For *Alopecurus myosuroides* in the greenhouse and fields, as well as the *Apera spica-venti* in the fields, the measurements were done on 3, 7 and 14 days after treatment (DAT). For *Apera spica-venti* in the greenhouse, the measurements were done on 3–4 DAT. Visual assessment of the herbicide efficacy for each marked plant was made 21 days after treatment. The herbicide resistance level was determined by the ratio of dead plants from each treatment, as proposed by Moss et al. [3,23]: “S” = sensitive, 100–81% mortality; “R?” = slightly resistant, 80–73% mortality; “RR”, resistant, 72–37% mortality; and “RRR”, strongly resistant, 36–0% mortality. Then, the plants were then classified as “sensitive” or “resistant”.

### 2.5. Data Analysis

Data were analyzed using R Studio [24], in order to determine if the herbicide treatments significantly affected *F_v_*/*F_m_*. We also examined whether the *F_v_*/*F_m_* value of sensitive and resistant plants can be differentiated under herbicide stress conditions. All the datasets were proved to be normally distributed by the Shapiro-Wilk test (*p* > 0.05). The homogeneity of variances was analyzed using Levene’s test (*p* > 0.05). Data from all the repeated experiments were gathered because the analysis of variance showed that there were no significant differences between the repeated measurements (*p* > 0.05).

## 3. Results and Discussion

### 3.1. Herbicide Effect Monitoring with the Chlorophyll Fluorescence Sensor

In Table 2, the average values of *F_v_*/*F_m_* of *Alopecurus myosuroides* in the greenhouse and field test, and the average values of *F_v_*/*F_m_* of *Apera spica-venti* in the field test are shown. All the plants (sensitive) with the herbicide treatment had lower *F_v_*/*F_m_* values than the untreated control plants after 3 DAT in the same location, and on the same date.

Visual assessment was conducted on 21 DAT in the greenhouse and the fields for all the CFI tested plants. The assessment results showed that all the populations of *Alopecurus myosuroides* and *Apera spica-venti* tested above were dead after treatment with pyroxsulam plus florasulam, or pinoxaden.

In Table 3, the results of the sensor and visual measurements of *Apera spica-venti* are presented. It shows that most herbicide-treated sensitive plants had lower *F_v_*/*F_m_* values than the untreated control plants, while the herbicide-treated resistant plants had similar *F_v_*/*F_m_* values to the untreated control ones. The accuracy of the measurement is 86.25% on pyroxsulam plus florasulam treated-plants, and 92.50% on pinoxaden-treated plants.

As mentioned in the sensor description, the chlorophyll fluorescence level is usually evaluated using the parameter *F_v_*/*F_m_*, which indicates the chlorophyll’s ability to transform the light energy into bioenergy, which can be used by plants for photosynthesis. In previous research, it has been found that the effect of PS II inhibitors on plants can be clearly identified with the chlorophyll fluorescence measurement because the inhibitor can directly bind on the D protein and interrupt the electron transfer chain of PS II [6,7,8,9]. Thus, more energy from the measuring light will be re-emitted as the chlorophyll fluorescence. As a result, the *F_v_*/*F_m_* values of the plants affected by the PS II inhibitors will markedly decrease.

ALS and ACCase inhibitors do not affect the photosystem itself. However, in this study the *F_v_*/*F_m_* variation in plants after herbicide treatment indicated that the PS II system could also be affected by the inhibitors of ALS and ACCase. Several previous studies may explain the mechanism of these inhibitors’ effect on PS II. When the plants are affected by ALS inhibitors, less valine, leucine and isoleucine are produced [25,26]. This results in a lower rate of synthesis of protein in plants that are sensitive to the mentioned herbicides [27]. Therefore, the electron transfer rate in these plants is reduced. This presents as lower PS II activity levels, and lower *F_v_*/*F_m_* values of the plants.

Comparing the chlorophyll fluorescence measurement results between plants with PS II inhibitors and ALS/ACCase inhibitors, the *F_v_*/*F_m_* induction of ALS/ACCase inhibitor-treated plants was not as intensive as in previous studies. In the soybean experiments by Li et al. [22], PS II inhibitors induced a more intensive effect on the PS II efficacy of the plants than the ALS and ACCase inhibitors. Considering the lower measuring accuracy in the greenhouse and the higher measuring accuracy in the field tests of the *Apera spica-venti* populations, for ALS/ACCase resistance detection in the field, a large scale of weed samples should be selected for measurement.

### 3.2. Abiotic Impacts on the Measurements

Considering all the untreated control plants of either the *Alopecurus myosuroides* or the *Apera spica-venti*, the *F_v_*/*F_m_* values of plants in the greenhouse were markedly higher than that of the plant in the fields. This indicates the environmental factors such as insufficient soil water content, sunlight and low temperature also have a stressful effect on the photo system of plants, as has been reported in many previous publications [28,29,30].

For most field trials, the measurements were carried out in late autumn or early spring, when the temperature in the fields was much lower than that in the greenhouses, but above 0 °C for sensible herbicide application. This could be the reason for the observed lower *F_v_*/*F_m_* values in the fields compared to the greenhouse. This was examined in a study of temperature stress on tomato plants [31], which found that there ws lower chlorophyll fluorescence emission in tomatoes grown under 22–26 °C compared to plants cultivated under 12 °C or over 33 °C. Therefore, lower *F_v_*/*F_m_* values were observed when plants were stressed by low or high temperatures. Even when there are different value ranges between measurements in the greenhouse and the field, the stress from low temperatures above 0 °C did not significantly affect the identification accuracy.

In fact, when the temperature is lower than freezing point, the frost can damage the chloroplast and weaken the photosystem [32]. This condition limits chlorophyll fluorescence sensing, which has been discussed by Wang et al. [19], who failed to obtain field measurements in temperatures below 0 °C. Besides, according to Zhou et al. [31], heat stress also affects the measuring results. As the herbicide is usually applied in spring and autumn, the heat stress experienced above 30 °C is unlikely to occur during the physiological reaction period of herbicide in in vivo plants.

Considering other abiotic stresses which might affect *F_v_*/*F_m_* measurement using the Weed-PAM sensor, drought stress is also a potential factor. This can decrease the CO_2_ availability and alternate the photochemistry and carbon metabolism [33]. A dissertation study by Wang showed that drought stress significantly reduced *F_v_*/*F_m_* in *Alopecurus myosuroides* seven days after exposure to water shortage, when the soil was extremely dry [34]. The *F_v_*/*F_m_* values of the plants under water shortage were extremely low with values of 0.1 compared to plants without stress (0.7). The stomata usually close during the initial stages of drought stress, resulting in increased water using efficiency [35]. However, under severe drought stress, dehydration of mesophyll cells takes place causing a marked inhibition of the basic metabolic processes of photosynthesis as well as a reduction in the water use efficiency of the plant [36]. However, in fact, most measurements with this sensor will be conducted soon after the sowing season or in the early spring, when the soil moisture is usually sufficient for crop growth. Thus, the extreme drought condition can be avoided when measuring.

Therefore, we conclude that this sensor can be used for real-time herbicide effect monitoring both in the greenhouse and in the field with suitable weather conditions including a temperature above 0 °C.

## 4. Conclusions

The fluorescence meter Weed-PAM is available to identify the effect of ALS and ACCase herbicides on PS II of weeds and crops with different populations and growing conditions. The system is able to be applied in both greenhouse and field environments. This technology will help farmers to ensure the herbicide application effect on weeds in the same growing season. This achievement can help farmers to avoid application failures that reduce crop yield.

## Figures and Tables

**Table 1:** Treatment herbicides and dose rates for greenhouse test of *Alopecurus myosuroides* and *Apera spica-venti*. Abbreviation: MoA, Mode of Action.

No.	Treatments	MoA	Water [L ha^−1^]	Herbicide [g ha^−1^ or L ha^−1^]	Additive [L ha^−1^]
1	control	-	200 L	-	-
2	pyroxsulam + florasulam	ALS	200 L	220 g	1 L
3	pinoxaden	ACCase	200 L	1.2 L	-

**Table 2:** Average value of *F_v_*/*F_m_* of *Alopecurus myosuroides*, *Apera spica-venti* and the Tukey’s HSD test results of treated and untreated plants. Significant codes due to *p* values: 0 ‘***’ 0.001 ‘**’ 0.01 ‘*’ 0.05 ‘.’ 0.1 ‘ ’ 1.

Species	Location	DAT	Treatments		
			Control	Pyroxsulam Plus Florasulam	Pinoxaden
*Alopecurus myosuroides*	Greenhouse	3	0.744 ± 0.007	0.703 ± 0.006 *	0.632 ± 0.034 **
		7	0.746 ± 0.006	0.659 ± 0.009 ***	0.532 ± 0.017 ***
		14	0.720 ± 0.010	0.672 ± 0.014 ***	0.478 ± 0.013 ***
	Renningen	3	0.701 ± 0.005	0.598 ± 0.006 ***	0.600 ± 0.018 ***
		7	0.703 ± 0.012	0.512 ± 0.008 ***	0.510 ± 0.012 ***
		14	0.720 ± 0.007	0.627 ± 0.003 ***	0.623 ± 0.011 ***
	Hohenheim	3	0.636 ± 0.009	0.613 ± 0.004 ***	0.603 ± 0.006 ***
		7	0.697 ± 0.006	0.604 ± 0.003 ***	0.646 ± 0.007 ***
		14	0.634 ± 0.011	0.463 ± 0.006 ***	0.562 ± 0.008 ***
*Apera spica-venti*	Renningen	3	0.696 ± 0.010	0.643 ± 0.008 ***	0.681 ± 0.012 ***
		7	0.701 ± 0.008	0.633 ± 0.006 ***	0.678 ± 0.008 ***
		14	0.714 ± 0.007	0.625 ± 0.013 ***	0.674 ± 0.007 ***
	Hohenheim	3	0.683 ± 0.004	0.624 ± 0.011 ***	0.661 ± 0.009 ***
		7	0.651 ± 0.007	0.596 ± 0.009 ***	0.623 ± 0.017 ***
		14	0.677 ± 0.005	0.597 ± 0.007 ***	0.624 ± 0.014 ***

**Table 3:** Average values of *F_v_*/*F_m_* of *Apera spica-venti* in the greenhouse and the Tukey’s HSD test results of treated and untreated plants. ALS: treatment with pyroxsulam plus florasulam; ACCase: treatment with pinoxaden; VA: visual assessment result; S: herbicide sensitive population; *: significant lower *F_v_*/*F_m_* value of the herbicide treated group than the untreated control group; ×, sensor and visual assessment results do not match.

No.	Control	ALS	VA	Verification	ACCase	VA	Verification
1	0.719 ± 0.014	0.695 ± 0.019 *	S		0.657 ± 0.017 *	S	
2	0.713 ± 0.009	0.718 ± 0.013			0.675 ± 0.012 *	S	
3	0.692 ± 0.012	0.697 ± 0.005	S	×	0.693 ± 0.011	S	×
4	0.691 ± 0.004	0.731 ± 0.005			0.667 ± 0.016 *	S	
5	0.712 ± 0.008	0.725 ± 0.006			0.668 ± 0.016 *	S	
6	0.708 ± 0.004	0.711 ± 0.005			0.687 ± 0.008 *	S	
7	0.706 ± 0.010	0.714 ± 0.009			0.672 ± 0.020 *	S	
8	0.701 ± 0.008	0.708 ± 0.007			0.699 ± 0.003		
9	0.720 ± 0.003	0.705 ± 0.011 *	S		0.657 ± 0.022 *	S	
10	0.715 ± 0.007	0.716 ± 0.010			0.640 ± 0.014 *	S	
11	0.712 ± 0.004	0.721 ± 0.004			0.665 ± 0.018 *	S	
12	0.732 ± 0.006	0.733 ± 0.004			0.641 ± 0.024 *	S	
13	0.724 ± 0.009	0.728 ± 0.003			0.693 ± 0.025 *	S	
14	0.693 ± 0.005	0.661 ± 0.007 *	S		0.660 ± 0.007 *	S	
15	0.765 ± 0.010	0.715 ± 0.036 *	S		0.679 ± 0.036 *	S	
16	0.738 ± 0.011	0.749 ± 0.010			0.675 ± 0.018 *	S	
17	0.717 ± 0.006	0.700 ± 0.006 *	S		0.685 ± 0.014 *	S	
18	0.735 ± 0.007	0.654 ± 0.015 *	S		0.701 ± 0.015 *	S	
19	0.659 ± 0.045	0.719 ± 0.016			0.574 ± 0.047 *	S	
20	0.689 ± 0.023	0.695 ± 0.004	S	×	0.700 ± 0.003	S	×
21	0.691 ± 0.006	0.705 ± 0.030	S	×	0.705 ± 0.036	S	×
22	0.725 ± 0.004	0.705 ± 0.013 *	S		0.654 ± 0.016 *	S	
23	0.718 ± 0.003	0.712 ± 0.006			0.692 ± 0.005 *	S	
24	0.713 ± 0.003	0.725 ± 0.017			0.692 ± 0.18 *	S	
25	0.728 ± 0.005	0.714 ± 0.007 *	S		0.712 ± 0.004 *	S	
26	0.718 ± 0.006	0.692 ± 0.004 *	S		0.699 ± 0.007 *	S	
27	0.724 ± 0.006	0.692 ± 0.013 *	S		0.713 ± 0.004 *	S	
28	0.721 ± 0.005	0.718 ± 0.016	S	×	0.705 ± 0.009 *	S	
29	0.723 ± 0.011	0.698 ± 0.004 *	S		0.696 ± 0.006 *	S	
30	0.732 ± 0.007	0.714 ± 0.005 *	S		0.690 ± 0.010 *	S	
31	0.735 ± 0.009	0.707 ± 0.011 *	S		0.688 ± 0.014 *	S	
32	0.723 ± 0.003	0.711 ± 0.007 *	S		0.700 ± 0.008 *	S	
33	0.733 ± 0.003	0.725 ± 0.004 *	S		0.714 ± 0.015 *	S	
34	0.720 ± 0.007	0.712 ± 0.002	S		0.706 ± 0.003 *	S	
35	0.712 ± 0.005	0.708 ± 0.005			0.689 ± 0.009 *	S	
36	0.700 ± 0.006	0.712 ± 0.006			0.694 ± 0.004		
37	0.719 ± 0.009	0.726 ± 0.004			0.669 ± 0.014 *	S	
38	0.720 ± 0.004	0.725 ± 0.004			0.696 ± 0.011 *		×
39	0.729 ± 0.005	0.713 ± 0.021			0.712 ± 0.008 *	S	
40	0.726 ± 0.008	0.732 ± 0.004			0.670 ± 0.015 *	S	
41	0.713 ± 0.006	0.722 ± 0.005			0.689 ± 0.007 *	S	
42	0.712 ± 0.011	0.709 ± 0.021			0.672 ± 0.005 *	S	
43	0.703 ± 0.027	0.732 ± 0.007	S	×	0.707 ± 0.006		
44	0.724 ± 0.004	0.732 ± 0.010			0.707 ± 0.011 *	S	
45	0.714 ± 0.003	0.724 ± 0.009			0.696 ± 0.006 *	S	
46	0.713 ± 0.008	0.717 ± 0.006			0.690 ± 0.004 *	S	
47	0.713 ± 0.012	0.738 ± 0.015			0.699 ± 0.007 *	S	
48	0.716 ± 0.013	0.715 ± 0.023	S	×	0.714 ± 0.030	S	×
49	0.725 ± 0.007	0.734 ± 0.002	S	×	0.708 ± 0.008 *	S	
50	0.719 ± 0.003	0.712 ± 0.005	S	×	0.709 ± 0.004 *	S	
51	0.720 ± 0.010	0.740 ± 0.011	S	×	0.723 ± 0.021	S	×
52	0.728 ± 0.004	0.735 ± 0.014			0.718 ± 0.003 *	S	
53	0.719 ± 0.004	0.716 ± 0.033			0.722 ± 0.019		
54	0.713 ± 0.010	0.728 ± 0.006			0.691 ± 0.007 *		
55	0.724 ± 0.007	0.714 ± 0.011 *	S		0.684 ± 0.008 *	S	
56	0.725 ± 0.006	0.731 ± 0.004	S	×	0.689 ± 0.014 *	S	
57	0.723 ± 0.003	0.715 ± 0.004 *	S		0.701 ± 0.006 *	S	
58	0.727 ± 0.008	0.730 ± 0.015			0.685 ± 0.030 *	S	
59	0.725 ± 0.005	0.729 ± 0.018			0.714 ± 0.023		
60	0.716 ± 0.018	0.737 ± 0.007			0.710 ± 0.015		
61	0.730 ± 0.005	0.707 ± 0.010 *	S		0.719 ± 0.003 *	S	
62	0.724 ± 0.007	0.696 ± 0.010 *	S		0.676 ± 0.017 *	S	
63	0.702 ± 0.003	0.710 ± 0.014			0.682 ± 0.015 *	S	
64	0.716 ± 0.004	0.720 ± 0.006			0.695 ± 0.003 *	S	
65	0.713 ± 0.009	0.739 ± 0.006			0.676 ± 0.017 *	S	
66	0.712 ± 0.012	0.716 ± 0.016			0.645 ± 0.020 *	S	
67	0.712 ± 0.005	0.727 ± 0.028			0.682 ± 0.013 *	S	
68	0.726 ± 0.006	0.717 ± 0.009			0.709 ± 0.010 *	S	
69	0.717 ± 0.003	0.729 ± 0.004			0.709 ± 0.004		
70	0.719 ± 0.004	0.722 ± 0.012			0.704 ± 0.010 *	S	
71	0.723 ± 0.003	0.722 ± 0.010			0.704 ± 0.014 *	S	
72	0.739 ± 0.013	0.737 ± 0.012			0.690 ± 0.025 *	S	
73	0.728 ± 0.006	0.746 ± 0.015			0.692 ± 0.010 *	S	
74	0.723 ± 0.004	0.705 ± 0.013 *	S		0.688 ± 0.010 *	S	
75	0.726 ± 0.011	0.732 ± 0.011			0.670 ± 0.019 *	S	
76	0.716 ± 0.007	0.699 ± 0.011			0.657 ± 0.017 *	S	
77	0.725 ± 0.009	0.720 ± 0.045	S	×	0.690 ± 0.011 *	S	
78	0.728 ± 0.012	0.722 ± 0.020			0.695 ± 0.008 *	S	
79	0.715 ± 0.006	0.712 ± 0.010			0.692 ± 0.016 *	S	
80	0.738 ± 0.014	0.708 ± 0.010 *	S		0.680 ± 0.016 *	S	
Accuracy Ratio				86.25%			92.50%
